# Identifying New Areas of Endemicity and Risk Factors for *Rickettsia conorii* subsp. *conorii* Infection: Serosurvey in Rural Areas of Romania

**DOI:** 10.3390/pathogens13090783

**Published:** 2024-09-11

**Authors:** Cristina Alexandra Cheran, Andreea Madalina Panciu, Claudia Doina Riciu, Iulia Maria Nedelcu, Diana Gabriela Iacob, Adriana Hristea

**Affiliations:** 1 Faculty of Medicine, Carol Davila University of Medicine and Pharmacy, 020021 Bucharest, Romania; andreea-madalina.firtat@drd.umfcd.ro (A.M.P.); iulia-maria.nedelcu@drd.umfcd.ro (I.M.N.); dianagiacob@gmail.com (D.G.I.); adriana.hristea@umfcd.ro (A.H.); 2 Department of Infectious Diseases, Bucharest Emergency Clinical Hospital, 014461 Bucharest, Romania; 3 “Prof. Dr. Matei Bals” National Institute of Infectious Diseases, 021105 Bucharest, Romania; laboratorvirusologie@yahoo.com; 4 Department of Infectious Diseases, Bucharest Emergency University Hospital, 050098 Bucharest, Romania

**Keywords:** *Rickettsia conorii*, Mediterranean spotted fever, serosurvey, Romania

## Abstract

Background: Mediterranean spotted fever (MSF) is an emerging tick-borne disease caused by *Rickettsia conorii* subsp. *conorii,* primarily prevalent in Mediterranean and Southern Europe. We aimed to evaluate MSF seroprevalence and risk factors in non-endemic rural areas of Romania. Methods: We conducted a serosurvey in five counties not under MSF surveillance by testing 459 serum samples from adult volunteers for specific IgG antibodies using ELISA. Participants answered a questionnaire regarding demographics and MSF risk factors. Results: The median age of the participants was 60 years and 329 (71.7%) were female. Overall, 64 (13.9%) samples tested positive for IgG anti-*R. conorii*, with rates ranging from 7.1% in Sibiu to 22.4% in Hunedoara. The median age of the positive individuals was 68 years, with a significantly higher seropositivity rate of 54.7% among those over 65 years (*p* = 0.01). Among those positive, 53 (82.8%) owned different household animals; 24 (37.5%) had daily contact with dogs, and 27 (42.2%) with livestock; 17 (26.6%) noted tick infestations of animals, and 23 (35.9%) reported tick bites. Conclusions: This study revealed an important seroprevalence of MSF in Romanian areas considered non-endemic, indicating an expansion of its geographical range, probably due to climate change, and emphasizing the importance of enhanced surveillance and diagnostic capabilities nationwide.

## 1. Introduction

Mediterranean spotted fever (MSF) is a re-emerging tick-borne disease. The risk of infection is notably higher in endemic regions, such as the Mediterranean basin, Southern Europe, parts of Africa, and in rural and suburban areas with abundant vegetation and favourable conditions for ticks. MSF cases have been documented in Italy, Portugal, Spain, France, Greece, Croatia, Türkiye, Switzerland, Bulgaria, and Romania, particularly during the warmer months when tick activity is at its peak [1]. 

The causative pathogen of MSF is *Rickettsia conorii* subsp. *conorii*, an obligate intracellular bacterium, slow-growing and Gram-negative, from the spotted fever group *Rickettsiales* (SFGR), transmitted to humans mainly by the brown dog tick (*Rhipicephalus sanguineus*) [2]. Ticks acquire *Rickettsia* species through transstadial transmission, where the pathogen is carried from one life stage to the next after a blood meal from an infected host or via transovarial transmission, where infected adult ticks pass the pathogen to their offspring. Transmission to humans occurs during tick bite, contact with arthropod infected secretions upon broken skin or mucosal membranes, or autoinoculation after unsanitary tick removal from animals [3]. It is noteworthy that other *Ixodidae* have been documented as a vector for *R. conorii,* such as species in the genus *Ixodes, Haemaphysalis, Dermacentor, Amblyomma,* and *Hyalomma* [4,5,6,7].

*Rickettsia conorii* is the second most frequent human infection within SFGR, after *R. rickettsii*, comprising 33% of the 66,133 human cases reported worldwide. Moreover, a systematic review on the global distribution of SFGR, which mapped population counts onto SFGR-suitable areas, predicted that *R. conorii* could potentially affect 3.7 billion people and cover 11.21 million km^2^, making it the second most risky infection within the SFGR after *R. felis* in terms of the at-risk population size and geographical range [4]. 

The clinical manifestations of MSF vary widely, ranging from mild, sometimes self-limiting, to severe illness, with vascular inflammation occurring in the central nervous system, lungs, heart, liver, pancreas, gastrointestinal tract, and kidneys. The black eschar (“tache noir”) at the site of the tick bite, despite being considered pathognomonic, can sometimes be overlooked or may not be present, leading to difficulties in differentiation from other febrile illnesses associated with a rash. This can lead to a delayed diagnosis and treatment, particularly in non-endemic areas where the disease is less familiar to healthcare providers, emphasising the importance of early identification and treatment to prevent complications and improve outcomes [3,8,9].

Romania is a country with a varied landscape and climate and a rich biodiversity in vertebrates and vectors, offering an ideal environment for ticks and tick-borne pathogens [5,10]. MSF presents a significant public health concern, particularly in regions where tick populations thrive due to the environmental changes and lack of protective measures [3,8], thus raising questions about the real extent of *R. conorii* transmission and its impact on human health within geographic areas where the infection is not under surveillance. 

Despite the existing serological evidence of *R. conorii* in Romania and the documentation of MSF cases since the first outbreak in 1931 in Constanta, involving 34 patients [11], the first identification of *R. conorii* using molecular tests was performed in 2016 in a *Rhipicephalus sanguineus* s.l. tick collected from a dog presented in the Veterinary Clinic of the Faculty of Veterinary Medicine of Bucharest [12]. Furthermore, a study conducted in Cluj-Napoca on ticks collected from urban wildlife identified the molecular presence of *R. conorii* in a co-infection with *Borrelia lusitaniae* in a questing *Haemaphysalis punctata* tick [6]. 

The prevalence of MSF in Romania remains underestimated in most counties, except for the Dobrogea and Bucharest region, considered the areas with the highest incidence and prevalence [11], as shown by a descriptive epidemiological study of MSF cases in Southern Romania from 2000 to 2008, identifying Constanta (with the highest incidence rate in the study, 44.2 per 100,000 inhabitants in 2001), Tulcea, and Bucharest as the most affected counties [13]. 

**Figure 1:**
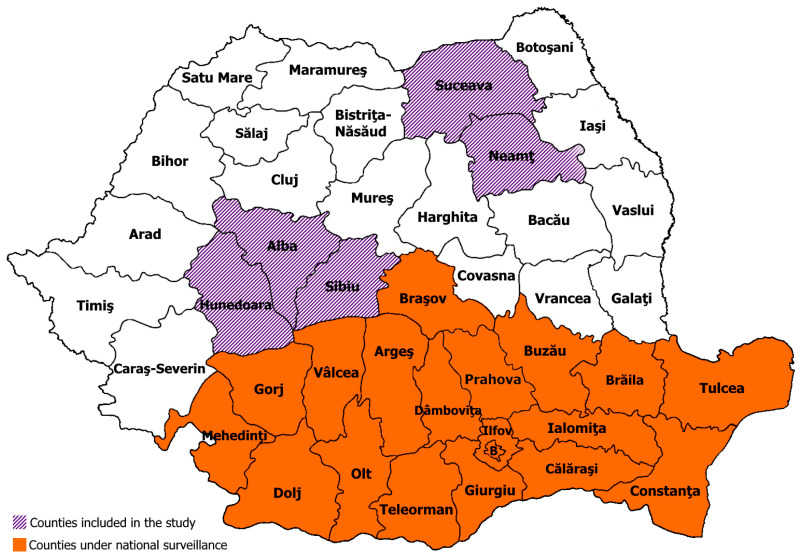
Map of Romania showing, in purple, counties included in the study and, in orange, counties under passive surveillance for MSF.

MSF is of mandatory declaration and under passive surveillance since 2000 in 19 counties of the Southern region of Romania, as shown in Figure 1. However, there is a lack of data regarding the annual number of cases of MSF in our country since 2017. According to the National Center for Surveillance and Control of Transmittable Diseases, the overall incidence of MSF ranged from 0.3 to 4.2 per 100,000 inhabitants between 2000 and 2017 [14]. Case definitions of MSF include possible cases based solely on clinical symptoms, probable cases that add epidemiological evidence to the clinical symptoms, and confirmed cases with positive antibodies against *R. conorii* detected by the indirect immunofluorescence assay (IFA). The laboratory criterion involves dynamic increases in IgM antibodies or a fourfold increase in IgG antibodies within paired sera, with samples collected 1–2 weeks apart [15]. 

Our objective was to evaluate the prevalence of antibodies against *R. conorii* and the risk factors for MSF in currently considered non-endemic areas of Romania, especially in the context of climate changes affecting our country. 

## 2. Materials and Methods

### 2.1. Study Design

We conducted a cross-sectional serosurvey using a convenient sampling strategy to detect serological evidence of *R. conorii* infection in population from rural areas of Romania, in regions outside the area known as being at risk. Our study included five localities from different counties: Albac (Alba), Romos (Hunedoara), Bazna (Sibiu), Zamostea (Suceava), and Ghindaoani (Neamt). 

The variation in participant numbers across sites was related to the number of individuals who volunteered to participate in the study.

### 2.2. Collection of Blood Samples

We collected a total of 459 blood samples during 2023, by joining a medical mobile unit offering a routine clinical examination and routine blood tests in remote rural areas. Considering natural exposure to ticks or animals in the daily life of the rural population, we included volunteers aged ≥18 years, living in rural areas of five Romanian counties. The participants signed the informed consent and answered a paper-based questionnaire addressing demographic data (age, gender, and profession), exposure to domestic or wildlife animals, tick infestation of the animals from their household, exposure to ticks, history of tick bite, and knowledge about tickborne diseases. The blood samples were collected in whole-blood vacutainer tubes and centrifuged after collection, frozen, and stored at −20 °C. The study was approved by the local Ethics Committee of the hospital where the serological study was performed (Protocol number C07704/30.06.2023).

### 2.3. Detection of Antibodies against Rickettsia conorii

Serum samples were tested for specific IgG antibodies against *R. conorii* antigen (Morroccan strain, ATCC VR-141) using the commercial VIRCELL IgG ELISA kit (VIRCELL, Granada, Spain), according to the manufacturer’s instructions, with optical density (O.D.) values set at >0.9 for positive control, <0.5 for negative control, and >0.55 and <1.5 for cut-off control. Each test run included these controls to validate the assay and kit. After calculating the mean O.D. for cut-off serum, results were expressed as an antibody index (AI), defined as the fraction of the sample O.D. and the cut-off serum mean O.D. multiplied by 10, with AI < 9.0 considered negative, AI = 9.0–11.0 as equivocal/borderline, and AI > 11.0 as positive. The optical density of the ELISA plates was read using an automated analyser, ELISA-EVOLIS (BIO-RAD, Hercules, CA, USA), at 450 and 620 nm. The sensitivity of the test is 85% (CI 75–92%), and the specificity is 100% (CI 95–100%) for IgG *R. conorii* ELISA, with no cross-reaction or interferences with *Coxiella burnetii* or *Legionella pneumophila* antibodies or antinuclear antibodies [16]. Equivocal samples were retested, as recommended by the manufacturer. 

### 2.4. Statistical Analysis

For statistical analysis, we used SPSS statistical package version 26.0 for Windows (SPSS, Chicago, IL, USA). The Chi-square test was performed to determine correlations among the various variables under examination, with statistical significance defined as a *p*-value less than 0.05. Non-parametric one-sample test was used to determine confidence intervals for study population. 

## 3. Results

We collected 459 serum samples from five localities in five counties in Romania outside the area under MSF surveillance (Figure 1). Demographic characteristics and data related to tick exposure are shown in Table 1. 

Most of the study population was represented by females in all the sites. The median age of the participants was similar across all sites except for one (Neamt), where the study population was older. People over 65 years old accounted for 40.3% of the study population. Among those aged under 65 years, the largest group of study participants, 119 (25.9%), were in the age group of 50–59.

We found a high variability for the presence of different conditions representing risk factors for tickborne infections. Thus, 95 (20.7%) participants had a profession at risk (farmers, hunters, forest rangers, and veterinarians), varying from <5% (Sibiu) to 30–45% in the two counties in the Moldova region (Suceava, Neamt). From a total of 352 (76.7) participants owning domestic animals, 144 (31.4%) reported observing tick infestations of their animals, ranging from 8 (11.4%) in Sibiu to 43 (46.2%) in Alba. 

In relation to the history of tick bites, 138 (30.1%) participants experienced tick bites in the past (Table 1). Of those with a history of tick bites, 122 (88%) reported one or few bites and 30 (21.7%) sought a medical consult for their tick bites. 

In total, 64 (13.9%) samples yielded positive results in IgG anti-*R. conorii* ELISA testing. We identified positive samples in all of the five counties included in the study, but the seropositivity varied between 7.1% in Sibiu to 22.4% in Hunedoara. 

Table 2 depicts the characteristics associated with the presence of antibodies against *R. conorii*. When comparing individuals with and without IgG antibodies against *R. conorii*, we found that an age over 65 years was significantly associated with a higher rate of seropositivity, with an OR of 1.97 (95% CI: 1.16–3.36). Specifically, the seropositivity rate among individuals under 65 years was 10.6%, while it was significantly higher (18.9%) in those aged 65 years and older.

Among individuals who tested positive for specific IgG antibodies against *R. conorii,* 53 (82.8%) owned different domestic animals, and 27 (42.2%) had daily contact with livestock. A history of tick bites was reported by 23 (35.9%) of individuals with positive samples compared to 115 (29.1%) of those with negative samples, but the difference was not significant (Table 2). 

Contact with dogs, considered a risk factor for MSF, was reported by 224 (48.8%) of the study population. Of these, 24 (10.7%) had specific antibodies. Interestingly, 200 (50.6%) of those with negative results vs 24 (37.5%) of people with positive results, OR of 0.74 (0.53–1.03), reported contact with dogs (Table 2). 

Almost two-thirds of the study population was represented by women. The median age of the 42 female participants with positive samples was 69 years (IQR 56,5–76). Among them, 8 (19%) were involved in a profession considered at risk for tick bite, 38 (90.5%) reported owning household animals, with 18 (42.9%) having daily contact with dogs, and 17 (40.5%) with livestock. Additionally, 12 (28.6%) reported tick bites. Furthermore, 15 (35.7%) indicated that they have been frequently engaged in mushroom picking.

## 4. Discussion

The findings of the current cross-sectional serosurvey provide new valuable information regarding the prevalence and distribution of MSF in Romania. Our study showed the presence of *R. conorii* IgG antibodies in subjects living in rural areas outside of the historically endemic region of Romania, in counties which are not under national surveillance for MSF. 

In our study, the overall seroprevalence was 13.9%, which is lower than that previously reported within our country (25.2%). Similar to other tick-borne diseases, MSF occurs in natural foci depending on the presence of both the vector and the reservoir and special ecological characteristics [17]. This focality leads to a highly variable seroprevalence between different geographical areas and poses a great challenge to obtaining a representative sample for a seroprevalence study [18]. The lack of testing for potential cross-reactivity with other *Rickettsia* species from the SFG is another limitation of our study.

Furthermore, the prior seroprevalence study conducted in Romania included only three historically significant foci of MSF from the South-Eastern region: Constanta, Tulcea, and Bucharest, with the highest prevalence at 32.8% in Constanta. A key finding of our study is that the seroprevalence in Hunedoara (22.4%) is comparable to that in Tulcea (22.9%) and higher than that in Bucharest (18.2%), both of which are known endemic regions in Romania [19]. Following Hunedoara, notable seroprevalence rates were also reported in our study in Suceava (16.7%) and Alba (10.8%). While Neamt has a higher median age and potentially increased occupational exposure, the lower positivity rate for IgG against *R. conorii* suggests that other factors might be at play. This could be related to differences in tick prevalence, exposure to specific tick species, or other protective factors within the population. 

Notably, the present study represents the first serological evidence of infection with *R. conorii* in the Hunedoara, Suceava, Neamt, Sibiu, and Alba counties. This highlights the extended geographical range of this infection and suggests that the environmental changes may be a causative factor. 

Research has confirmed a link between changes in climate variables and an increasing incidence of tick-borne diseases worldwide. Warmer temperatures throughout the year favour the expansion of the geographical range of ticks, and alter tick behaviour by accelerating their activity, feeding, and reproduction, thereby introducing new pathogens in areas where they were previously absent [20,21,22,23,24,25]. 

According to the 2023 climate report by the National Meteorological Administration, Romania has been experiencing rising temperatures, with 2023 being the warmest year on record since 1961. The maximum temperature reached 42 °C, and the national average temperature was 11.4 °C, marking substantial positive deviations of 2.3 °C from the 1981–2010 median and 1.8 °C from the 1991–2020 median, indicating a clear trend of increasing temperatures over recent decades, consistent with the global warming trend [26].

Furthermore, multiple studies conducted in Romania revealed an expanding habitat of ticks, new tick species, and tick-borne pathogens being described within this geographic area in the last 20 years [27,28,29,30,31,32]. In a study that evaluated ticks collected from animal hosts across Romania, several species of SFG Rickettsiales were identified. Of particular note, *R. conorii raoultii* was found in Alba, and *R. helvetica* in both the Suceava and Alba counties, while *R. monacensis* was detected in Suceava [10]. 

Another important observation from our data is the association between the age above 65 years and the higher seroprevalence for MSF. Our study included a significant number of participants aged above 65 years (40.3%), related to the progressive aging of the rural population in our country. The median age of the subjects with positive serum samples was 68 years vs 59 years in people without antibodies against *R. conorii.* These findings are consistent with previously reported data from a seroprevalence study conducted in Southeastern Romania (Bucharest, Constanta, Tulcea) in 2009, where the most affected age group was >60 years, followed by the age group of 21–30 years. This study included 301 participants with a larger range of age (median age of 39 years), compared to the population of our study (median age of 60 years). Serum samples were collected from asymptomatic individuals during the seasonal evolution of MSF and tested using the ELISA method, revealing an overall seroprevalence of 25.2% [19]. 

Additionally, the most recent data about the evolution of MSF in Romania, published by the National Institute of Public Health in 2017, reported a total of 102 suspected cases, of which 87 (85.3%) were serologically confirmed. Similar to our study, the most affected age group was 65–74 years. The highest incidence was recorded in Tulcea county (12.3 per 100,000 inhabitants), followed by Constanta (4.3 per 100,000 inhabitants), with significantly lower values in the rest of the counties under surveillance. While, in 34.5% of the cases, the source of infection was represented by infected dogs, contact with tick-infested animals was noted in 6.9% of the cases [14]. Similarly, contact with dogs was found in our study in about one-third (37.5%) of the cases, but the rate of tick infestation of their household animals was higher (26.6%). 

Dogs serve as the primary reservoir for *R. sanguineus* and their infestation with ticks infected with *R. conorii* is a significant predisposing factor for MSF [33,34]. It is noteworthy that *R. sanguineus* also targets a wide range of wild and domestic animals, exposing them to and potentially serving as reservoirs for *R. conorii.* Evidence of antibodies against *R. conorii* in pigs, donkeys, cattle, goats, sheep, rabbits, mules, and horses suggests their potential role in the epidemiology of MSF [34,35].

Notably, 82.8% of positive cases in our study reported having different domestic animals in their households and contact with livestock was more frequent in positive samples (42.2%) compared to negative ones (33.4%), albeit not being statistically significant (*p* = 0.171). 

The intricate interactions of climate and environmental changes, socio-demographic factors, and animal and tick exposure highlight the need for integrated surveillance systems and targeted public health interventions to effectively manage and mitigate the risks associated with tick-borne diseases like MSF.

## 5. Conclusions

The current serosurvey revealed the presence of *R. conorii* IgG antibodies in individuals living in rural areas outside of the typically endemic regions, expanding the known geographical risk area of MSF in Romania. Consistent with previous findings, our study revealed a significantly higher seroprevalence rate among participants over the age of 65. The identification of new areas with serological evidence of MSF indicates the potential for broader endemicity and challenges in disease management and prevention. In the context of current climate changes in Romania, these findings emphasize the importance of broader surveillance and enhanced diagnostic capabilities in healthcare settings all over the country, in order to promptly identify and manage MSF cases.

## Figures and Tables

**Table 1:** Study population characteristics and seropositivity rates by county.

Study Population and Counties, N (%)	Alba N = 93 (20.3)	Hunedoara N = 107 (23.3)	Neamt N = 93 (20.3)	Sibiu N = 70 (15.4)	Suceava N = 96 (20.9)	Total N = 459 (95% CI)
Male sex, N (%)	33 (35.5)	26 (24.3)	19 (20.4)	20 (28.6)	32 (33.3)	130 (28.3) (24.2–32.7)
Female sex, N (%)	60 (64.5)	81 (75.7)	74 (79.6)	50 (71.4)	64 (66.7)	329 (71.7) (67.3–75.8)
Median age (years) (IQR)	59 (50–66)	59 (51–69)	70 (59–75.5)	54.5 (48–68)	58 (48–67.75)	60 (50–71)
Profession at risk ^1^, N (%)	15 (16.1)	7 (6.5)	42 (45.2)	2 (2.9)	29 (30.2)	95 (20.7) (17.1–24.7)
Domestic animals in household ^2^, N (%)	79 (84.9)	85 (79.4)	65 (69.9)	31 (44.3)	92 (95.8)	352 (76.7) (72.5–80.5)
Contact with dogs, N (%)	42 (45.2)	43 (40.2)	39 (41.9)	18(25.7)	82 (85.4)	224 (48.8) (44.1–53.5)
Contact with livestock, N (%)	53 (57)	25 (23.4)	34 (36.6)	6 (8.6)	41 (42.7)	159 (34.6) (30.3–39.2)
Tick infestation of their household animals, N (%)	43 (46.2)	35 (32.7)	20 (21.5)	8 (11.4)	38 (39.6)	144 (31.4) (27.2–35.8)
Mushroom collector, N (%)	32 (34.4)	26 (24.3)	16 (17.2)	0	59 (61.5)	133 (29) (24.9–33.4)
Tick bite history, N (%)	49 (52.7)	40 (37.4)	25 (26.9)	13 (18.6)	11(11.5)	138 (30.1) (25.9–34.5)
ELISA *R. conorii* positive samples, N (%) (95% CI)	10 (10.8) (5.3–18.9)	24 (22.4) (14.9–31.5)	9 (9.7) (4.5–17.6)	5 (7.1) (2.4–15.9)	16 (16.7) (9.8–25.6)	64 (13.9) (10.9–17.5)

^1^—profession at risk = hunter, forest ranger, and farmer; ^2^—domestic animals = dogs, cats, cows, sheep, goats, horses, pigs, and birds; CI—confidence interval; IQR—interquartile range.

**Table 2:** Comparative characteristics of the study population associated with the presence versus the absence of IgG *R. conorii* antibodies.

Study Population and Counties, N (%)	Positive Samples N = 64	Negative Samples N = 395	Total Samples N = 459	OR (95% CI)	*p*-Value
Male sex, N (%)	22 (34.4)	108 (27.3)	130 (28.3)	1.25 (0.86–1.82)	0.2
Female sex, N (%)	42 (65.6)	287 (72.7)	329 (71.7)	0.9 (0.74–1.1)	0.2
Median age (IQR) (years)	68 (54.2–76)	59 (49–69)	60 (50–71)	-	-
Age > 65, N (%)	35 (54.7)	150 (38)	185 (40.3)	1.97 (1.16–3.36)	0.01
Profession at risk ^1^, N (%)	16 (25)	79 (20)	95 (20.7)	1.25 (0.78–1.99)	0.3
Domestic animals in household ^2^, N (%)	53 (82.8)	299 (75.7)	352 (76.7)	1.09 (0.96–1.23)	0.2
Contact with dogs, N (%)	24 (37.5)	200 (50.6)	224 (48.8)	0.74 (0.53–1.03)	0.05
Contact with livestock, N (%)	27 (42.2)	132 (33.4)	159 (34.6)	1.26 (0.91–1.73)	0.1
Tick infestation of their household animals, N (%)	17 (26.6)	127 (32.2)	144 (31.4)	0.82 (0.53–1.27)	0.3
Tick bite history, N (%)	23 (35.9)	115 (29.1)	138 (30.1)	1.23 (0.86–1.77)	0.2
Mushroom collector, N (%)	22 (34.4)	111 (28.1)	133 (29)	1.22 (0.84–1.77)	0.3
History of recreational activities in nature, N (%)	1 (1.6)	9 (2.3)	10 (2.2)	0.68 (0.08–5.32)	0.7

^1^—profession at risk = hunter, forest ranger, and farmer; ^2^—domestic animals = dogs, cats, cows, sheep, goats, horses, pigs, and birds; CI—confidence interval; IQR—interquartile range.

## Data Availability

The original contributions presented in the study are included in the article, further inquiries can be directed to the corresponding author.
